# Engaging patient-led rare disease organizations to advance research – through the lens of Bardet–Biedl syndrome

**DOI:** 10.1242/jcs.264320

**Published:** 2025-10-27

**Authors:** Timothy Ogden, Bendert de Graaf, Tonia Hymers

**Affiliations:** ^1^ BBS Foundation, PO Box 663 Unionville, PA 19375, USA; ^2^ Bardet Biedl Syndroom Stichting, Keulvoet 12, 8266KK Kampen, The Netherlands; ^3^ BBS UK, 43 Balton Way, Dovercourt, Harwich, Essex, CO12 4UP, UK

**Keywords:** Ciliopathies, Rare genetic diseases, Bardet–Biedl syndrome, Patient-led rare disease organization, Collaboration

## Abstract

For researchers and clinician-scientists, forging partnerships with patient-led rare disease organizations can be a challenge. Patient-led rare disease organizations often operate quite differently to research and medical institutions, large private or public funding organizations, and pharmaceutical or biotechnology companies, leaving researchers and clinician-scientists uncertain about how, when and where to engage for mutual benefit. However, the value of reciprocal engagement can be immense, paying dividends in new research directions, accelerating existing research, facilitating access to funding and achieving success in translation. Most importantly, it can improve the lives of individuals with disease. In this Perspective, we will explore the value of engaging and collaborating with patient-led rare disease organizations through the lens of a rare syndromic ciliopathy – Bardet–Biedl syndrome (BBS) – for which patient-led organizations exist in multiple countries. We explain what researchers should know about how rare disease organizations operate, discuss examples of successful engagement between researchers, clinician-scientists and patient-led organizations, and review the ‘do's and don't's’ of successful collaboration.

## Teaser

**Summary:** This Perspective discusses how effective collaboration between researchers and patient-led rare disease organizations is necessary, possible and powerful, building on the authors' experiences leading patient groups for the rare ciliopathy Bardet–Biedl syndrome.

## Introduction

Bardet–Biedl syndrome (BBS) is a rare autosomal recessive ciliopathy, a class of diseases caused by defects in the formation or function of cilia, hair-like cell protrusions with a variety of important cellular and physiological functions (Forsythe and Beales, 2013). The prevalence of BBS is estimated at one in 160,000 to one in 100,000, rising to one in 15,000 or less in areas with a founder effect (e.g. Newfoundland) or consanguineous populations (Forsythe et al., 2018; Moore et al., 2005; Farag and Teebi, 1989). BBS was first described more than 100 years ago in separate reports by Bardet (1920) and Biedl (1922). The first attempt at systematizing clinical diagnosis for BBS came in 1999 (Beales et al., 1999). Based on a review of patient records, Beales et al. describes ‘primary features’, including rod-cone dystrophy, polydactyly, obesity and renal anomalies, as well as ‘secondary features’, or less common features, including hepatic fibrosis, development delay, ataxia and diabetes. Notably, the Beales et al. (1999) study came about via collaboration with a BBS patient group based in the UK, and the patient group coordinator was a co-author. Historically, clinical diagnosis was usually ascertained through ophthalmologists or nephrologists who were either observant enough to note features outside of their specialty, such as polydactyly and obesity, or pushed by persistent patients and caregivers seeking a unitary explanation for a wide variety of symptoms. Clinical diagnosis remains difficult even today because of the high degree of variability in the presence and severity of symptoms, both between and within families, and in the evolution of symptoms over a patient's lifetime.

The first gene locus linked to BBS was identified in the early 1990s by studying consanguineous Bedouin populations (Kwitek-Black et al., 1993); the first gene associated with the syndrome was subsequently reported in 2000 (Slavotinek et al., 2000; Katsanis et al., 2000). By 2010, 15 such genes had been identified via classic genetic approaches. Advances in sequencing technology have enabled the discovery of at least 12 more genes (27 total) linked to the syndrome, accounting for ∼80% of the clinically diagnosed population, with pathogenic variants in the ten most commonly affected genes accounting for ∼70% of cases and pathogenic variants in the two most commonly affected genes (*BBS1* and *BBS10*) accounting for ∼30% of the population (Forsyth and Gunay-Aygun, 2003, updated 2023). Overall, BBS is highly genetically and clinically heterogeneous, with largely unknown genetic modifiers influencing clinical presentation.

The molecular understanding of BBS was transformed when impaired cilia were first linked to the syndrome with the discovery of the protein Bardet–Biedl syndrome 8 (BBS8, also known as TTC8) (Ansley et al., 2003). Next, landmark studies (Nachury et al., 2007; Loktev et al., 2008; Jin et al., 2010) described the assembly and function of the BBSome, a multiprotein complex constructed of seven BBS proteins with a major role in trafficking of proteins in and out of the cilium. An additional three BBS proteins were found to serve as chaperonins important for BBSome assembly and function. These discoveries positioned BBS as a ciliopathy, deepening our understanding of the syndrome and the role of cilia in human health and disease.

The development of next-generation sequencing has dramatically transformed access to clinical genetic testing for BBS, with numerous benefits. In 1999, the reported average age at diagnosis was 9 years (Beales et al., 1999). Based on our experience with newly diagnosed families who have reached out to patient-led groups, diagnoses are now often made earlier, allowing families to access support and information sooner, whereas older individuals are receiving long-awaited explanations for their symptoms. A broader range of phenotypes, including mild or atypical cases (Deitch et al., 2024), has also emerged, expanding our understanding of the variability of the syndrome and improving epidemiological estimates. However, with greater appreciation for clinical variation and expanded access to genetic testing, we are also seeing – particularly in the USA – increasing confusion regarding the application of clinical and genetic diagnostic criteria. Higher rates of both diagnosis and false-positive misdiagnosis highlight the need for better education, clearer diagnostic guidelines and integrated care pathways.

BBS patient groups first emerged in the 1980s and early 1990s as sufficient numbers of individuals were clinically diagnosed. A group of families in the USA with family members who had received a diagnosis of BBS began gathering at conferences of the Foundation Fighting Blindness, under the heading of ‘other rare genetic causes of blindness’, and then with the support of an ophthalmologist clinician-researcher. Around the same time, a group also formed in the UK, initiated by a group of families with students diagnosed with BBS at a school for the visually impaired who started working with Contact a Family (https://contact.org.uk/), a national charity dedicated to connecting families with rare diseases. Eventually, led by Dr Phil Beales and the UK patient group, the UK established the first dedicated BBS clinical service (https://bbsuk.org.uk/bbs-clinics-service/) in the world, significantly improving care coordination and enabling affected families to connect with others more easily. By the 2010s, patient-led organizations (with varying levels of formality) were operating in North America, the UK, France, The Netherlands, Italy, Norway and several other countries. Today, these organizations are actively engaged with more than 1500 families across the world. Patient-led BBS groups are instrumental in funding or providing other support for clinical registries in the US, UK, The Netherlands and more, supporting research protocols and the development of research materials, disseminating information, facilitating research recruitment, and providing support information and other services through in-person meetings and events or online platforms. The largest Facebook group for BBS patients and families has more than 2500 members worldwide, reflecting the importance of peer support and community engagement.

In this Perspective, we discuss how effective collaboration between researchers and patient-led rare disease organizations is necessary, possible and powerful, both for advancing fundamental research into disease mechanisms and most importantly, for improving the lives of patients. Building on our experiences leading BBS patient groups (Box 1), we explain the goals, functions and operations of rare disease organizations, share examples of successful and respectful engagement between researchers, clinician-scientists and patient-led organizations, and review the ‘do's and don't's’ of successful collaboration.

Box 1. About the authors and their patient-led groups Tonia Hymers has been the Operations Manager of Bardet–Biedl Syndrome UK (BBS UK; https://bbsuk.org.uk/) for 8 years. Tonia, who has an adult child diagnosed with BBS, has volunteered for BBS UK for 28 years and previously served as a Family Support Officer for 7 years. BBS UK, which includes over 700 BBS families, aims to promote the welfare of persons who have BBS and advance the education of medical and educational professionals and the public about BBS.

Bendert de Graaf is a Founder of the Netherlands-based Bardet Biedl Syndroom Stichting (https://www.bardetbiedlsyndroom.nl/), a patient group of around 150 BBS families. Bendert helped establish the foundation following his child's diagnosis with BBS in 2016 and has served as its Chair for 10 years, working to build a platform to help parents, doctors, researchers and patients connect and access information in order to increase awareness of and research into BBS.

Timothy Ogden is the founder of the Bardet–Biedl Syndrome Foundation (BBSF; https://www.bardetbiedl.org/), a US-based patient group serving North America that includes around 1000 BBS families. Timothy, whose now adult child was one of the first in the world diagnosed prenatally, was elected President of the former BBS Family Association in 2012, leading the effort to formally establish the Foundation and expand its resources and outreach. He has led the organization for 13 years, recently transitioning from a volunteer to a paid role.

## The value of engagement and collaboration

From a patient perspective, the massive progress in understanding primary cilia in the past 15 years has been both exciting and frustrating. For those whose diagnoses predate the identification of BBS genes, much less the proteins, complexes and metabolic functions involved, this was the future we had long hoped for. However, this progress in basic science has not yet translated into meaningful changes in clinical care or patient outcomes. There is currently only one approved pharmaceutical intervention for BBS, which is indicated for just one non-universal feature of the syndrome – Imcivree (setmelanotide), a melanocortin 4 receptor (MC4R) agonist, has been approved for treatment of hyperphagia in the US and UK (Markham, 2021). Gene therapy for arresting retinal degeneration is inching forward for at least two of the most common BBS-linked genes (e.g. Hsu et al., 2022). The situation across syndromic ciliopathies is similar – although basic science has flourished, translational research and improvements in clinical care remain limited and are often restricted to symptomatic management. We believe that the most important steps to continue to facilitate progress in basic research and in clinical care are increasing engagement and collaboration between researchers and patient groups.

### Influencing priorities

Despite significant advances in our fundamental understanding of primary cilia, numerous crucial basic science questions remain unresolved. From the perspective of individuals affected by BBS and based on discussions with members of other ciliopathy communities, who report similar concerns, we have identified several issues that in our view represent urgent research priorities.

#### Why does BBS vary so widely in terms of symptoms and severity?

As in many other ciliopathies, the clinical presentation of BBS exhibits a large degree of heterogeneity, even when caused by the same gene or the same variant. In some cases, siblings can have nearly opposite manifestations of retinal degeneration (rapid versus slow) and kidney disease (requiring a transplant versus mild chronic kidney disease). How much of this variation is due to genes, due to variants, due to oligogenic factors or due to environmental factors?

#### Can we predict the highly variable penetrance, progression and severity of symptoms?

A specific concern for many families is learning about the likely pace of retinal degeneration or progression of kidney disease. For example, what biomarkers correlate with how quickly the retinas of an affected individual degenerate, or the likelihood of significant progression of chronic kidney disease? Similar to the previous question, what role might oligogenic or environmental factors play in accelerating or protecting against progression of various symptoms?

#### What is the function of each BBS protein in diverse cell and organ types during all stages of life and how can we comprehensively study these functions?

Many fundamental questions regarding ciliary protein function remain open. What are the non-ciliary roles of BBS proteins and other proteins involved in cilia formation and function, and do their functions differ during development, homeostasis and regeneration? For example, some BBS proteins localize to the nucleus of cells. Do ciliary proteins participate in additional cellular pathways not previously reported, and if so, could these pathways be leveraged for gene-agnostic therapies?

#### What explains the inconsistent overlap between symptoms of syndromic ciliopathies?

Part of answering this question involves ascertaining the cause of disease for the ∼20% of people who are clinically diagnosed with BBS but do not have an identified genetic cause. These cases might be explained by misdiagnosis between syndromic ciliopathies, such as Alstrom, Bardet–Biedl, Joubert and Senior–Loken syndromes, by variants of unknown significance (VUS) in known BBS genes or by pathogenic variants in unknown genes. Ultimately, understanding the overlap between syndromic ciliopathies should shed light on the complex signaling cascades whose disruption leads to symptoms and open multiple pathways to potential treatments.

### Driving hypotheses

Within the context of these high-priority questions for patient groups, the role of patient engagement becomes not only valuable but essential. Basic research is driven by hypotheses, and the experience of rare disease patients can be a vital accelerator to forming hypotheses. In our view, meaningful progress in understanding and treating complex, highly variable rare diseases, including BBS, is not possible without sustained engagement and partnership with patient-led organizations. These groups play a vital role in both basic and translational research, serving as catalysts, connectors and collaborators for scientific discovery. Indeed, many important insights have emerged from patient conversations and have unmasked the truly systemic effects of the primary cilia defects seen in BBS. We have seen these developments play out in formal and informal interactions, between researchers and patient groups, several times.

For example, the recognition of anosmia as a feature of BBS began with questions raised at a UK patient conference. After several patients or caregivers noted an impaired sense of smell, researchers developed a protocol to test for anosmia at the next patient gathering and in clinical visits, culminating in a paper documenting a high prevalence of anosmia in BBS individuals (Kulaga et al., 2004). Similarly, at a US patient gathering, a family member asked whether Legg–Calvé–Perthes disease (LCPD), a degenerative hip condition caused by disrupted blood supply to the femoral head, was a feature of BBS. The researchers present accurately answered that it had not ever been described as part of BBS. However, four other families also present immediately noted that the individual with BBS in their family had also been treated for LCPD. Higher rates of orthopedic complications, including LCPD and Blount's disease, which affects tibia growth, have now been observed in the Clinical Registry Investigating Bardet–Biedl Syndrome (CRIBBS; https://www.bbs-registry.org/).

Over the past decade, as genetic testing has proliferated, it has become increasingly clear that the early literature on BBS captured only the individuals with severe symptoms. Moreover, it provided a biased understanding of the various associated symptoms: because researchers and clinicians were looking for individuals with a body mass index (BMI)>40, chronic kidney disease and/or cognitive delays, the diagnosed population was biased toward higher prevalence and severity of these symptoms. Today, patient organizations are growing to include middle-aged or older people recently diagnosed through genetic testing, whose symptoms other than retinal degeneration were not severe enough to merit follow-up with a specialist earlier in life and who might not have met clinical diagnostic criteria. Their inclusion is reshaping how we define BBS and, in our view, it should also be reshaping research programs and best practices for clinical care.

Engaging with sufficiently large patient populations across ages, developmental stages and symptom profiles in rare disease is essentially impossible without partnering with patient organizations who have done the hard work of building trust and connections with patients and their families. The same logic applies to translational research, which can and should be driven by the experiences of patient populations. Importantly, regulatory agencies like the Food and Drug Administration (FDA) in the US, European Medicines Agency (EMA) in the European Union and the Medicines and Healthcare Products Regulatory Agency (MHRA) in the UK are embedding Patient and Public Involvement (PPI) in their processes, requiring that the design, evaluation and approval of therapies is informed by patient perspectives. In doing so, they reinforce the principle that patient organizations are not optional collaborators but essential translational research partners. From a functional perspective, there are many examples of translational research failures that have emerged from a lack of understanding of lived experience of patients managing symptoms and the trade-offs between symptoms and medication side effects, as well as failures in development of meaningful clinical endpoints. Table 1 shows examples of how patient organizations can contribute to translational research efforts.

**Table 1.** Collaborative opportunities with patient-led groups for translational research

Research stage	Roles for patient organizations
Priority setting	Identifying knowledge gaps, co-defining research questions
Study design	Advising on feasibility, acceptability, measurable and meaningful patient outcomes
Funding applications	Emphasizing the impact of research
Ethics	Highlighting hidden burdens and risks
Conduct	Supporting recruitment, managing attrition, supporting qualitative data gathering and analysis
Dissemination	Supporting plain language summaries and use of accessible platforms, advocating for uptake

### Funding

In addition to driving research questions and patient recruitment, rare disease patient organizations are increasingly essential funders and fundraisers. Some act as strategic partners, improving the quality and effectiveness of grant applications, whereas others offer direct financial support and have the resources to fund and sustain significant infrastructure. The BBS Foundation (US; https://www.bardetbiedl.org/) funded the creation of CRIBBS in 2014 and has supported it since. Today, CRIBBS is the largest clinical registry for BBS, with more than 750 patients enrolled and more than 350 having completed multiple annual interviews. The support by the BBS UK group of the BBS Clinics Service has led to the UK registry surpassing 650 enrollees, with some patients contributing more than 15 years of longitudinal data. Even patient-led rare disease organizations with limited budgets typically have a big advantage over large institutional funders – the ability to pivot and move quickly. For instance, at the start of the SARS-CoV-2 pandemic, BBS patient organizations were able to fund small grants within a few weeks to keep animal model programs from being terminated.

In parallel, large institutional funders are also increasingly recognizing the value of patient voices and involvement in influencing research. These funders now often require patient representatives to be included either by grant applicants in research planning or in grant review panels. This shift has led to a growing emphasis on patient involvement, for instance, in the form of patient boards in research hospitals in the UK and The Netherlands. For rare disease researchers, initiating collaborations with patient organizations early in the research development process can significantly strengthen funding applications and enhance the relevance and impact of proposed work. With research funding becoming increasingly competitive, partnerships with patient-led organizations are no longer optional – they are a differentiator that elevates an interesting or promising funding application and, even more importantly, increases the likelihood that research translates into real-world benefit.

## Successful engagement

Recognizing the value in collaborating with patient-led rare disease organizations is an important step. However, it begs the question of how researchers can begin new collaborations or improve existing collaborations with patient organizations. Here, we present three necessary aspects of successful collaboration – respectful listening, alignment of expectations and balancing of priorities, and breaking down barriers.

### Respectful listening

Although it might seem unnecessary to preface the term ‘listening’ with words like ‘respectful’ or ‘meaningful’, our experience tells us it is not only appropriate but essential. We have experienced positive collaboration, being a respected partner in research planning, being treated as experts in lived experience and cooperation on shaping questions that mattered both to science and to the patient community – but we also have directly experienced in the recent past being told that patient input was ‘nice’ but would risk the scientific integrity of the project, or of being told that our invitation to participate in a research meeting was because “we have to invite patients” and then being directed to remain silent during the ‘important’ parts of the discussion. How patients are involved in research matters just as much as whether they are involved. Therefore, successful collaboration begins with respectful listening.

Respectful listening requires an accurate understanding of who is in the conversation. A persistent barrier to listening and ultimately to collaboration is that many researchers lack experience or knowledge of patient-led organizations generally, much less knowledge of rare disease organizations or of a specific organization. In the absence of familiarity, assumptions might fill the gap. Researchers might assume that patient groups lack scientific understanding or interest in research, or that ultra-rare disease organizations might be similar to organizations for more ‘common’ rare diseases, like cystic fibrosis or polycystic kidney disease, with significantly greater resources and expertise. These assumptions can lead to miscommunication, misplaced expectations and ultimately, failed collaborations.

We have made the case that patient-led organizations can and should be essential collaborators, but that does not mean that every rare disease patient organization is prepared to engage in the same way. In this section, we outline key dimensions of patient-led organizations that can help researchers begin to understand their diverse goals, capacities and capabilities (see Table 2 for examples). Although similarities exist, there are no fixed correlations – each organization occupies its own space on these axes, shaped by its community, history, resources and mission.

**Table 2.** Example patient-led syndromic ciliopathy organizations, highlighting differences in governance, staffing, funding and function

Organization	Website	Governance	Staff	Source of funding	Functions
BBS Foundation (USA)	https://www.bardetbiedl.org/	Incorporated non-profit, independent board	Some staff, mostly volunteer	Private fundraising	Supporting, Advocating, Connecting, Fundraising, Investing
BBS UK	https://bbsuk.org.uk/	Incorporated non-profit, independent board	Staffed	Government support, private fundraising	Supporting, Advocating, Connecting, Fundraising, Investing
Bardet Biedl Syndroom Stichting (The Netherlands)	https://www.bardetbiedlsyndroom.nl/	Incorporated non-profit, independent board	Volunteer	Government support, private fundraising	Supporting, Advocating, Connecting, Fundraising, Investing
BBS Germany	https://www.pro-retina.de/pro-retina/fachbereiche-und-arbeitskreise/mitgliedervernetzung-und-kommunikation-beratung/arbeitskreis-bardet-biedl-syndrom-bbs	Unincorporated, operated as part of another non-profit, Pro Retina Deutschland	Volunteer	–	Supporting, Connecting
A Race Against Blindness (USA)	https://araceagainstblindness.org/	Incorporated non-profit, single-family	Staffed	Private fundraising	Fundraising, Investing
Joubert Syndrome & Related Disorders Fund (USA)	https://jsrdf.org/	Incorporated non-profit, independent board	Volunteer	Private fundraising	Supporting, Advocating, Connecting, Fundraising
Alstrom Syndrome International (USA)	https://www.alstrom.org/	Incorporated non-profit, independent board	Staffed	Private fundraising	Supporting, Advocating, Connecting, Fundraising, Investing

#### Governance

Understanding the governance of a patient organization is necessary to understand who the organization is speaking for and how decisions are made. The most important factor is whether the organization is legally incorporated and in what form (e.g. a non-profit or foundation, a membership organization, or other form, depending on the country and legal framework). Legal incorporation typically brings requirements for transparency, accountability and financial reporting, which in turn can build trust with the broader patient community, and especially with funders and regulators.

Other important considerations exist. These specifically include whether the organization is governed by a single family, by a few families, or has a governance structure that includes a broader set of affected families. Some organizations are democratically structured membership organizations, with elected boards that reflect a more-diverse patient population. Others might be governed by the board itself (rather than members), with specific requirements for board members with lived experience or expertise, including medical or research experts. Of course, some organizations have boards that are more ‘for show’ and are controlled by a single individual or family. Much of what researchers need to know about representativeness, governance, authority and decision-making can be gleaned from the website of an organization, but there remains a role for engaging and listening.

#### Staffing

Many patient-led rare disease organizations are entirely ‘staffed’ by volunteers. Others have a fully professionalized staff with multiple layers of management and full-time research or policy teams, and many fall somewhere in between. When engaging with an organization, it is important to understand the staff resources available – but do not equate limited or volunteer-only staffing with a lack of scientific expertise or professionalism. The volunteer staff at some organizations can include CEOs, scientists, clinicians, biotech venture capitalists, lawyers and other deeply knowledgeable professionals.

#### Source of funding

Funding models for patient-led organizations often reflect regional differences. American organizations tend to be entirely privately funded, whereas European organizations might have direct government support, funding from public grants or be integrated into national health or research systems. Both public and private sources of funding can come with restrictions on how, where and whether funds can be used to support research. Moreover, beyond the private versus public funding distinction is the capacity of the organization to attract or influence additional funding. An organization might have limited funds, but still maintain strong relationships with individual philanthropists, corporate sponsors or institutional donors.

#### Functions

The most important and most variable dimension of rare disease patient organizations is the collection of functions that a particular organization carries out. Some organizations are strictly focused on research and others on supporting patients and families, but most are engaged in a variety of functions. Ensuring that collaborative conversations begin by setting out a clear understanding of the primary functions of an organization and its capacity is crucial, as it helps set realistic expectations, fosters mutual respect and lays the groundwork for truly productive and sustainable collaboration. We classify the functions of an organization into five categories – supporting, awareness and advocacy, connecting, fundraising and investing.

First, supporting is the basic function of providing information and community to families affected by a rare disease. This can be the most visible function of an organization but can potentially mislead people in the scientific community to assume that the organization is not interested in or not capable of collaborating on research. For example, the images and content designed to appeal to and welcome a family that has just been diagnosed will necessarily be simplified and can be mistaken for a lack of interest in basic science, but keep in mind that this public aspect of our work is necessary to build trust and connection with our communities and facilitate quality patient participation over the long term.

Second, awareness and advocacy are the work of influencing anything from policy, clinicians, school officials, workplace practices and more to improve lives and reduce barriers for patients.

Third are the connecting functions, which have the aim to build bridges between affected families and clinical care, research and scientific advances. These often take the form of producing summaries of recent research, hosting talks by expert clinicians or scientists or generating other content that helps patient families understand their condition and what research is being conducted.

Fourth is fundraising, which includes all efforts to bring in funds to support the other functions. Notably, messaging around fundraising might not always align closely with content created under the connecting function. Just as simplified messages can be used to present an ‘open door’ to new patients and families, effective fundraising messaging might dramatically simplify the science, or reflect the most optimistic view, even if the organization is more realistic about the operational constraints of research.

Fifth, investing is the work of cultivating direct or indirect funding or support for basic research, translational research, clinical trials, clinical practice, centers of excellence or standards of care, etc. This can include, for instance, serving on advisory boards that influence research funding or participating in co-development of research funding proposals.

### Aligning expectations and balancing priorities

Listening is, of course, not a one-way street – successful collaboration requires all parties to engage in respectful listening to each other. That is the basis of the second aspect of successful collaboration – aligning expectations and balancing priorities. Some rare disease patient organizations that do not engage in the connecting or investing functions might lack familiarity with the operational realities of research (funding mechanisms and cycles, academic structures, institutional constraints, regulatory requirements, etc.). In such cases, the patient organizations might have unrealistic expectations of what researchers can do, even when the interest of the researchers in collaboration is strong. Therefore, it is important to build on respectfully listening to patient organizations by engaging in respectful communication in both directions. Respectful listening should be the basis for adjusting vocabulary, information and expectations in conversations, and asking about the patient organization's positive and negative experiences with research is one way of showing respect.

Important outcomes from respectful listening include developing a shared understanding of what the patient organization can deliver, what parts of a project it can contribute to and areas where additional support might be needed. When expectations are misaligned, clear communication about goals, timelines and constraints of researchers can help establish clarity and understanding. In our experience, most patient organizations approach collaboration with a strong sense of realism and purpose, with a focus on impact rather than idealized outcomes. Still, effective communication with patient communities requires empathy, clarity and an appreciation of the emotional landscape in which families are living. In this regard, researchers sometimes struggle to strike the right tone between overly optimistic projections that raise unrealistic expectations, and overly cautious assessments that shatter hope. Additionally, researchers might have difficulty maintaining effective communication at appropriate intervals. Here, we relate some anecdotes from the BBS community that illustrate these challenges.

In one example, every 5 years for the last 20 years at least, patient organizations have heard from ophthalmology researchers that gene therapies for retinal degeneration are less than 10 years away. Although we are very encouraged by the progress toward a Phase 1 clinical trial of gene therapy targeting *BBS1*, there is now understandable skepticism underlying this excitement among the patient community. Another example occurred when a prominent researcher in the field was approached after a conference by grateful parents, eager to say thank you for scientific efforts that could ultimately benefit their son. The researcher, unaware and apparently uninterested to learn that one of the parents was also deeply experienced in research, responded by condescendingly explaining the research process and timelines and telling the couple that they should “just get used to” the idea that their son would lose his sight. Although the response was accurate, and arguably better than giving false hope, it squandered a huge amount of goodwill that could have been the basis of future collaboration and engagement.

These examples underscore a simple but powerful point – how information is communicated matters just as much as what is communicated. When it comes to setting and aligning expectations, the goal should be communication that does not pander or condescend to the patient community. The key questions important to the patient organization and community are almost always about understanding how a research collaboration will ultimately benefit patients. That benefit need not be immediate or direct, just clear. Clarity is the only basis for balancing priorities in the collaboration. Again, the patient organization will generally want to ensure that a collaboration includes effective communication of any research insights back to the patient community. Therefore, project outputs that benefit or are directed to the patient community should not be left as afterthoughts. These outputs can be as simple as producing plain language summaries for patient group use, inviting patient groups to review and provide feedback on draft manuscripts, or attending patient group meetings to interact directly with the community.

### Breaking down barriers

Finally, effective collaboration in rare disease research requires researchers and clinicians to break down barriers between each other and work across traditional boundaries. Research and funding are competitive, and ambition is welcome; however, when researchers do not collaborate with other researchers, the cost to patients is immense. Limiting other researchers' access to patients or data might make a grant application more competitive in the short term, but slower progress means irrevocable damage to lives. We have encountered multiple instances of researchers being hesitant to collaborate, whether by declining to speak at events hosted by other researchers, not sharing data, protocols or resources, or not pursuing joint efforts that would unquestionably benefit the patient community. To be clear, patient organizations are not immune from the factors that undermine collaboration between groups. Some patient organizations refuse to collaborate with other organizations for patients with the same, or related, disease.

These decisions, even if grounded in practical concerns, can have real consequences for patient communities who are most often looking for unity and urgency in advancing care and understanding. For common diseases, there has often been funding specifically for collaboration and the creation of open-access databases, such as the Catalogue of Somatic Mutations in Cancer (COSMIC; https://cancer.sanger.ac.uk/cosmic; Forbes et al., 2008), and large funders have the ability to require and enforce collaboration. This is rarely the case in rare disease, where patient groups are usually dependent on researchers ‘doing the right thing’. This does happen – for instance, researchers at the Institute of Molecular Genetics in Prague, Czechia, created the open access ‘dataBBaSe’ of basic data on nearly 1000 BBS patients by extracting and systematizing data from published papers and case reports (https://bardet-biedl.img.cas.cz/; Niederlova et al., 2019).

Another barrier to break down is that between researchers, clinician-scientists and clinicians (either specialist or non-specialist). Especially in syndromic ciliopathies, which share a high degree of heterogeneity in symptom prevalence and severity, connecting research to actual patient experiences is essential for validating translation from cellular and animal models to human patients. Fortunately, breaking down this barrier is now easier than ever. Registries exist for a wide variety of rare diseases. As noted above, the BBS Foundation maintains CRIBBS with more than a decade of data, and the UK has a long-running clinical registry produced by the BBS Clinics Service. Registries have also been created in The Netherlands and Norway (Rustad et al., 2025) among other countries. In fact, the significant barrier currently is not the absence of clinical registries, but the absence of researchers using the data that has been collected. Furthermore, including clinician-researchers and clinicians in any collaboration signals that researchers are willing to balance the priorities of scientific discovery and translation to improve patient lives. Fostering collaboration across disciplines and institutions can be challenging, but doing so builds trust and accelerates impact. For rare diseases, that impact can be profound.

## Conclusion

Successful and impactful collaboration between basic science researchers, translational researchers and patient-led rare disease organizations is possible. In the BBS community, we have seen it and benefited from it. We have also seen other rare disease organizations, like the Rory Belle Foundation for NARS1 disease, the Gorlin Syndrome Alliance, the Alliance to Cure Cavernous Malformation and Congenital Hyperinsulinism International (e.g. Pasquini et al., 2025) benefit. We only plead for more researchers to prioritize collaboration with patient groups.

In this Perspective, we have focused on the practical benefits, barriers and opportunities involved in engaging patient-led rare disease organizations in disease research. We have not yet touched on the most fundamental reason for more collaboration – it is simply the right thing to do. Collaborating with patient groups shows respect for the essential dignity of people affected by rare disease. They are experts, not subjects. They deserve research that is conducted with them, not just on them. They deserve a voice in the allocation of resources toward what is most important to them, not just to what is most interesting academically or viable commercially.

We close by reiterating the most fundamental principle of successful collaboration – respectful listening. We plead with ciliopathy researchers the world over to listen to patients, listen to patient groups and lay the groundwork for changing research and changing outcomes for rare diseases.
